# Bridging Scales: a Hybrid Model to Simulate Vascular Tumor Growth and Treatment Response

**Published:** 2023-06-09

**Authors:** Tobias Duswald, Ernesto A.B.F. Lima, J. Tinsley Oden, Barbara Wohlmuth

**Affiliations:** a CERN, Geneva, Switzerland; b School for Computation, Information, and Technology, Technical Universtity of Munich, Germany; c Oden Institute for Computational Engineering and Sciences, The Universtity of Texas at Austin, United States of America; d Texas Advanced Computing Center, The Universtity of Texas at Austin, United States of America

**Keywords:** Vascular tumor growth model, Angiogenesis, Combination therapy, Agent-based model, Hybrid model, 3D tumor simulation

## Abstract

*Cancer* is a disease driven by random DNA mutations and the interaction of many complex phenomena. To improve the understanding and ultimately find more effective treatments, researchers leverage computer simulations mimicking the tumor growth *in silico*. The challenge here is to account for the many phenomena influencing the disease progression and treatment protocols. This work introduces a computational model to simulate vascular tumor growth and the response to drug treatments in 3D. It consists of two agent-based models for the tumor cells and the vasculature. Moreover, partial differential equations govern the diffusive dynamics of the nutrients, the vascular endothelial growth factor, and two cancer drugs. The model focuses explicitly on breast cancer cells over-expressing HER2 receptors and a treatment combining standard chemotherapy (Doxorubicin) and monoclonal antibodies with anti-angiogenic properties (Trastuzumab). However, large parts of the model generalize to other scenarios. We show that the model qualitatively captures the effects of the combination therapy by comparing our simulation results with previously published pre-clinical data. Furthermore, we demonstrate the scalability of the model and the associated C++ code by simulating a vascular tumor occupying a volume of 400*mm*^3^ using a total of 92.5 million agents.

## 1. Introduction

According to the WHO [1], cancer is one of the deadliest diseases worldwide and was responsible for one out of six fatalities in 2020. In the same year, officials registered 18 million new cases, roughly matching the entire population of the Netherlands. The sheer number of people suffering from cancer and the accompanying protracted fight against the disease drew many scientists into cancer research. Experimentalists and theoreticians alike strive to foster the understanding of tumor growth, disease progression, and different treatment protocols. United in their goal to battle cancer and improve the life quality of patients, experimentalists gather quantitative data on cancerous systems, while at the same time, theoreticians explore mathematical models for the disease, i.e., attempting to predict its evolution and reaction to treatment.

Mathematical cancer models usually belong to one of the following three categories: (1) models based on ordinary differential equations (ODEs), (2) models based on partial differential equations (PDEs), and (3) models based on discrete cell representations, which we refer to as agent-based models (ABMs). In 2016, Oden and co-workers [2] reviewed these approaches and embedded them into the wider context of the predictive, computational sciences, and the associated data-generating experiments. Earlier work from Byrne [3] and Beerenwinkel [4] documented the progression of the field and offer a great introduction to the topic.

Each of these modeling approaches involves strengths and weaknesses. For instance, ODE models are comparatively cheap to compute but fail to resolve spatial structures. PDEs incorporate spatial information but are significantly more expensive to implement computationally. Further, they employ homogenized tumor properties (i.e., from tumor cells to cell densities), which may benefit more extensive tumor simulations but limits their ability to resolve effects on a cellular scale. This scale is best described with ABMs resolving the individual tumor cells and allowing a natural way to include cellular information. Unfortunately, the computational costs can quickly get out of hand. In 2019, Metzcar and coworkers [5] reviewed ABMs and their application in theoretical cancer research. Their work offers an excellent overview of the state of the art and the wide range of models leveraged by scientists.

Mathematical tumor models tend to consider simplified scenarios and, ABMs in particular, often focus on small simulations because of the computational costs. While simple models should generally be preferred [6], cancer thrives from complex interactions. To better understand them, the complexity must, of course, be captured by the mathematical models. In the experimental literature, researchers working on *in vitro* drug screening have long realized that, for instance, 3D cell cultures and tumor spheroids better match *in vivo* studies than flat, 2D cultures [7] and that the complex tumor microenvironment has a strong influence on the tumor development [8, 9, 10, 11]. Thus, it is of great interest to replicate the system’s complexity *in silico* to test and improve the current understanding with computational models [12]. However, the complexity poses new challenges as it requires significant software development effort upfront before a specific problem in a complex environment can be studied.

In this work, we present a novel hybrid model (ABMs + PDEs) simulating vascular tumor growth and the response to therapy combining Doxorubicin and Trastuzumab in 3D. Our model consists of three major components: (1) a set of PDEs governing the diffusive dynamics of the nutrients, the vascular endothelial growth factor (VEGF), and cancer drug compounds, (2) an off-lattice, center-based ABM with spherical agents for the tumor cells governed by a cell cycle, and (3) another off-lattice, center-based ABM with cylindrical agents describing the vasculature and sprouting angiogenesis. The PDEs and ABMs are coupled in both ways, i.e., the continua influence the agents and vice versa. The vasculature supplies nutrients and treatment drugs, which are consumed by the tumor cells; in contrast, tumor cells secrete VEGF triggering vascular growth via sprouting angiogenesis. Moreover, the tumor cells interact via two-particle forces. To overcome the previously outlined limitations, we base our implementation on the highly-efficient ABM simulation platform BioDynaMo [13, 14], which enables our C++ application code to scale seamlessly from single-core machines to modern compute nodes with hundreds of threads. The entire source code of the project is available, together with a Docker container and bash scripts to reproduce the findings.^1^

While adopting ideas from previous research, most notably [15, 16, 17, 18, 13], this work represents several significant advances. First, we extend the previous ABMs [16, 17, 18] to a treatment scenario accounting for two different cancer drugs and move from 2D to 3D. Second, we show a novel way to model the vasculature and angiogenesis in a general ABM context and couple it with PDE models. Third, we show that the model qualitatively describes several aspects of tumor dynamics and captures the expected characteristics of the combination therapy as hypothesized by Jain [19] and experimentally investigated in [20]. Lastly, we demonstrate that the code can handle tissue-relevant sizes by simulating a 9 × 9 × 9*mm*^3^ large volume hosting up to 92.5 million agents over 27 days. Computationally, this significantly exceeds that in previous work.

We first introduce the biological mechanisms of vascular tumor growth, the considered cancer drug treatment, and the associated preclinical study [20] in Section 2. We proceed by detailing the mathematical model in Section 3 and devote Section 4 to discussing our parameter choices. In Section 5, we run the fully coupled model demonstrating the model’s ability to simulate vascular tumor growth and treatment by comparing the simulation results to the pre-clinical study. We scale our simulation to tissue-relevant scales in Section 6. We critically review our approach and address shortcomings in Section 7. Additionally, Appendix A displays the data used in Section 6, Appendix B gives an overview of all model parameter, and Appendix C explains our approach for statistically mimicking the initial vasculature for the tissue scale.

## 2. Preliminaries and Model Framework

In this work, we present a hybrid model simulating the vascular growth of a tumor and its decline under treatment. This section establishes the biological and medical background to understand the model’s components and reviews the literature. We begin with summarizing the most important biological concepts of vascular tumor growth and point the reader to related mathematical literature. In Section 2.2, we sketch the mechanism of action of the two cancer drugs considered by our model, Doxorubicin and Trastuzumab, and outline why a treatment combining both may excel in efficacy in contrast to current practice [19]. The preclinical study supporting this hypothesis [20] is presented in Section 2.3 and provides the most relevant data for this study.

### 2.1. Vascular Tumor Growth and Mathematical Models

**Figure 1:**
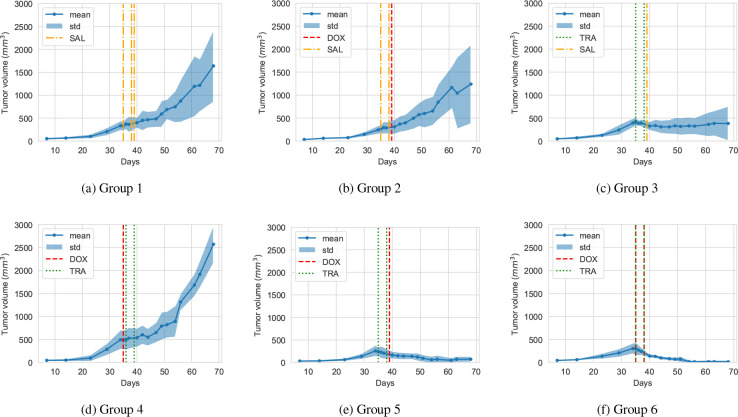
Mean and standard deviation of the tumor volume, measured over 70 days, of six different treatment groups: (a) group 1, (b) group 2, (c) group 3, (d) group 4, (e) group 5, and (f) group 6. The vertical lines indicate the day when each treatment was delivered (Doxorubicin (DOX), Trastuzumab (TRA), Saline (SAL)). Data taken from [20] and [57. Tab. 1 and 5].

Cancer is a disease evolving on a cellular scale; on the most fundamental level, seemingly random mutations of the cell DNA occur during the regular cell cycle. These DNA changes trigger abnormal behavior, mainly affecting cell proliferation and mobility. Typically, cancerous cells replicate quicker than healthy cells enabling them to locally out-compete the normal cells for resources. However, increased proliferation and mobility are only two among many phenomena that differentiate tumor cells from regular cells. In a seminal series of papers [21, 22, 23], Hanahan and Weinberg identified the *hallmarks of cancer*, i.e., specific properties that either tumor cells or populations thereof show in contrast to normal tissue due to the altered DNA. They describe ten hallmarks and four further candidates as of 2022 [23, Fig. 1]. While these hallmarks characterize the tumor cells on small scales, Nia et al. [24] linked the hallmarks to macroscopic properties, which they called the *physical traits of cancer*. These traits encompass stress, pressure, stiffness, and the complexity of the tumor microenvironment. The hallmarks and physical traits of cancer form a solid basis for the theoretical investigation of cancerous systems using mathematical tools [25].

Among the ten hallmarks, *inducing and accessing vasculature* is particularly important for the present study. If a local population of tumor cells grows, a commonly observed pattern is that it drains the locally available energy resources, e.g., oxygen and glucose, and creates a deadly, hypoxic environment for all cell types. The tumor cells enter a hypoxic state and secrete signaling substances such as VEGF to attract new vasculature, an observation usually attributed to Folkman [26]. The existing vasculature reacts by forming new sprouts that grow towards the hypoxic region to supply oxygen and rescue the dying cells. This process, called sprouting angiogenesis, forms a central component of this study. We note that there are alternative mechanisms to increase the tumor’s vascular density. However, sprouting angiogenesis is usually dominant, and consequently, we focus on it in the present work (see [27, 1.2.1])

As explained in the introduction, cancer dynamics are typically modeled with ODEs, PDEs, ABMs, or combinations thereof. An excellent summary of ODE methods can be found in Benzekry’s work [28]. PDE based models leverage diffusive terms [29, 30] or a phase field description [31, 32, 33]. More recently, models involving fractional diffusion dynamics have been considered [34]; i.e., diffusion processes that deviate from the traditional Flick’s law. ABMs have recently been reviewed by Metzcar [5]. It is common practice to combine the three approaches, e.g., using an ABM with cell internals modeled with an ODE system while diffusing substances are modelled with PDEs [16, 35].

Similar modeling paradigms have also been used to model the phenomena of (sprouting) angiogenesis. Villanova [27] presents an introduction to the topic in his Ph.D. thesis by summarizing different modeling approaches and explaining the biological background. In his research [36, 37], he combines a discrete model for the tip cells with a phase field model following them and classifying regions as being vasculature or not, an approach similar to [38]. Fritz and co-workers [39] describe a complex, coupled PDE model with a network growth algorithm considering the vasculature’s statistical features. For more general reviews of angiogenesis models, we refer the reader to [40, 41], but for the present work, purely agent-based angiogenesis models are at the center of attention. Arguably one of the most important works in this regard has been carried out by Bentley et al. [42, 43]. They describe an initial blood vessel by points located on a cylinder connected via mechanical springs and reacting to external substances. Each point resembles an agent acting independently, forming sprouts and predecessors to vessels. Perfahl and co-workers [44] modelled the vasculature as a chain of spherical agents connected via springs showing similarities to our approach. In contrast, Phillips et al. [18] model angiogenesis in a 2D setting resolving the individual cells of the vessels modelled as tip and stalk cells. Their cancer model shares significant features with ours, but the angiogenesis module is conceptually different. Furthermore, we use the evolving vasculature to model the supply of Doxorubicin and Trastuzumab, two drugs discussed in the next section.

### 2.2. Doxorubicin and Trastuzumab

We consider a treatment protocol involving the two well-known cancer drugs: Doxorubicin (DOX) and Trastuzumab (TRA). The U.S. Food and Drug Administration approved these drugs in 1974 and 1998, respectively, and they routinely find use in clinical applications. DOX is an *anthracycline* frequently used in chemotherapy, popular because of its high efficacy in fighting many different types of cancer. In typical treatment scenarios, DOX is injected into the patient’s veins, from where it spreads through the body and, ultimately, begins interacting with the cells. Effectively, DOX interrupts the DNA duplication by a process referred to as *intercalation* [45, 46]. Once cells fail to duplicate their DNA, they trigger safety mechanisms, often leading to the cell’s death[47, 48]. For more information on DOX and its effects on cells, we refer the reader to [49, 50, 51, 52] and references therein.

TRA is a *monoclonal antibody* and, thus, is more specific in its therapeutic action than DOX. In general, a monoclonal antibody is an antibody that only binds to a specific molecular structure (e.g., a protein). After binding, the antibody induces an immune reaction targeting its binding partner, which may depend on the monoclonal antibody and the binding partner. Historically, monoclonal antibodies had much success in cancer therapy [53]. TRA specifically binds to the so-called *human epidermal growth factor receptor type-2* (HER2) located at the surface of some tumor cells. HER2 is often over-expressed in dangerous breast cancer variations (20–30%). The associated pathways lead to increased proliferation and, thus, tumor formation. When TRA binds to HER2, it inhibits proliferation and reduces survival. Moreover, there is evidence that TRA shows anti-angiogenic properties; i.e., it stops the formation of new blood vessels and prunes and regularizes the exiting tumor vasculature [54, 55]. For an in-depth literature review, see [56].

While both drugs have proven effective in fighting cancer, they may also have severe side effects. For instance, DOX has been linked to cardiotoxicity, neurological disturbances, and many other maladies (see references in [50, Section 3]). TRA is less harmful, but side effects still occur [56, Toxicity]. *Combination therapy* strives to combine different drugs into one therapy strategy such that the drugs enhance each other’s anti-tumor tumor properties while minimizing their toxicity, i.e., damage to the patient. In 2001, Jain [19] suggested a new paradigm for combining anti-angiogenic therapies with regular tumor treatment. He argued that anti-angiogenic drugs could be used to regularize the tumor vasculature, allowing it to deliver other anti-cancer drugs more effectively. For the case at hand, TRA would regularize the vasculature improving its supply properties. Afterward, lower doses of DOX may be sufficient to eradicate the tumor cell population. In the present work, we provide a computational model designed to illustrate this effect and to compare it to preclinical data introduced in the next section.

### 2.3. In vivo Experiments for Combination Therapy

Sorace et al. [20] tested Jain’s hypothesis [19] in a pre-clinical *in vivo* study. They injected HER2+ breast cancer cells (BT474, ATCC) into the murine subjects and observed the tumor evolution over 70 days. They split the 42 murine subjects into six different treatment groups:

- Group 1: control group, treated with saline,
- Group 2: treated with DOX only,
- Group 3: treated with TRA only,
- Group 4: first treated with DOX, subsequently with TRA,
- Group 5: first treated with TRA, subsequently treated with DOX,
- Group 6: simultaneously treated with DOX and TRA.

All 42 animals remained untreated for 35 days and showed similar disease progression. Once the treatment started, Sorace and coworkers observed significant differences in tumor volume over time between the groups. The observations are displayed in Fig. 1, which shows that the tumor volume grows exponentially before the treatment begins. Furthermore, the treatments of groups 2 and 4 are observed to be ineffective. For group 3, we observe stagnation, and for groups 5 and 6 a significant decline in tumor volume. The data of these experiments were published in [57, Tab. 1 and 5] together with a calibrated ODE model. We merged the pre-treatment stages of the six groups into a separate dataset given in Tab. A.2 in Appendix A These data, specifically the pre-treatment stage and the groups 2, 3, 4, and 5, play a fundamental role in assessing the quality of our hybrid model later in the results and discussion sections. We now shift our attention to the core of this work: the hybrid model.

## 3. The Hybrid Model

Our model incorporates the evolution of the tumor mass, its nutrient and blood supply, and the effects of the therapy in one comprehensive hybrid model. The tumor mass is described with an agent-based model composed of individual, spherical tumor cells independently progressing in their cell cycle but dependent on the concentration of external substances. The cells interact via two-particle forces. The blood vessels are modeled with individual, cylindrical agents managed in a tree-like structure, i.e., each agent has precisely one predecessor and either one or two successors. The developing vasculature delivers the nutrients and drug compounds to the cells but also reacts to the local VEGF gradient. Four substances are modeled as scalar fields: nutrients, VEGF, DOX, and TRA. All four substances obey reaction-diffusion equations and are coupled to the ABM via source and sink terms proportional to regularized δ-distributions marking the agents’ locations. Hence, the coupling between PDEs and ABMs is bidirectional. We implemented the model in C++ based on the highly efficient BioDynaMo framework [13, 14].

In the following subsections, different parts of the model and their interactions are described. We begin with the tumor cells, their cell cycle, and their interaction forces in Section 3.1. We continue with the blood vessels and explain the rules governing the dynamics of angiogenesis in Section 3.2. The equations governing the scalar fields are detailed in Section 3.3. After discussing the separate components of the model, we couple them in Section 3.4.

### 3.1. Tumor cell

Our model describes the tumor on the cellular scale; i.e., each tumor cell is explicitly modeled as a spherical agent with stochastic behaviors. In the simulation, a tumor cell is a C++ object with various attributes. Focusing on the most relevant examples, a tumor cell is characterized by (1) a unique ID, (2) its position $\vec{x}$ in the three dimensional space, (3) its nuclear, physical, and action radii ($r_{n},\;r_{p},\;r_{a}$), (4) its cell state s, and (5) an internal clock tracking the time since the last state transition $\Delta t_{s}$ to model the time-dependent phases of the cell cycle. All but the cell state s are real, possibly vector-valued numbers.

The cell state s is a categorical variable taking values s∈{Q,SG2,G1,H,D}, Q is the quiescent state in which the cell is idle and no special events occur. SG2 and G1 denote the proliferative cell states. In SG2, the cell duplicates its inner components and prepares for cell division. The volume-preserving cell division marks the transition from SG2 to G1. In G1, the cells grow until they reach their natural size. The states H and D denote the hypoxic and dead states, respectively.

The different cell states form the core of the tumor growth model. The transitions between them depend on the values of the four continua - the nutrients, VEGF, DOX, and TRA. We denote them as $u_{n},\;u_{v},\;u_{d}$, and $u_{t}$, respectively. The second basic component of the tumor model is the force model consisting of the repulsive and adhesive forces governing the cell-cell interaction. In what follows, we describe the stochastic model underlying the state transitions and detail the forces and their algorithmic computation afterward. The model of the cell cycle and the forces are, in part, based on previous work [15, 16, 18, 17].

#### 3.1.1. Cell cycle

**Figure 2:**
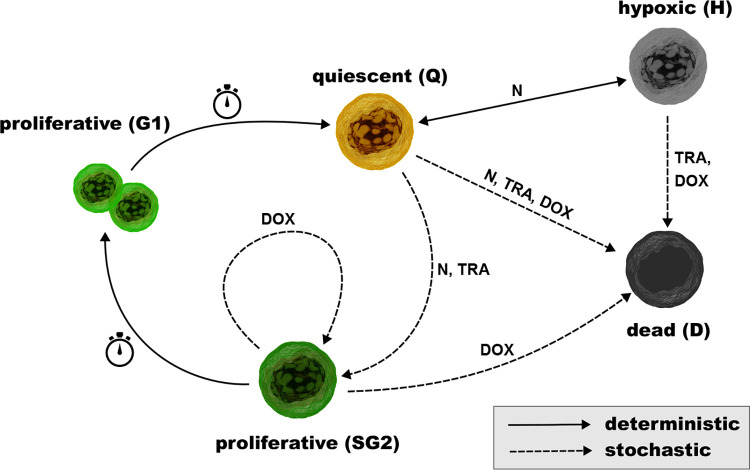
The cell cycle for the tumor cells. Deterministic and stochastic transitions are indicated by solid and dashed lines, respectively. The arrows indicate the direction of the transition. Transitions depending on the concentration of the nutrients (N), DOX, or TRA are labeled accordingly. Transitions solely depending on an internal clock are label with a stopwatch. Cell representation: *Cancer cell from the DataBase Center for Life Science (DBCLS) distributed under Creative Commons Attribution 4.0 International license (modified). Stopwatch: Stop Watch from SimpleIcon distributed under Creative Commons Attribution 3.0 Unported.*

The progression of a tumor cell in its cell cycle depends on the local substance concentrations but not on the surrounding cells. The state transitions are governed by stochastic as well as deterministic rules. Given the five states (Q,SG2,G1,H,D), our cell cycle allows transitions Q→SG2/H/D,SG2→G1/D,G1→Q, and H→Q/D. In its entirety, the cell cycle is best represented graphically as depicted in Fig. 2.

We first focus on the three deterministic transitions: SG2→G1,G1→Q, and Q→H. The first two transitions simply require the cell to spend a given time in the cell state assuming that the time for the cells to duplicate their internals and their growth is fixed. For SG2→G1 and G1→Q, the times are $T_{SG2}$ and $T_{G1}$, respectively. The transition from Q to H depends on the nutrient concentration, i.e., if the concentration falls below the hypoxic threshold, $u_{n}^{H}$, the cells transition from Q to H; if the concentration raises above $u_{n}^{H}$, the cells transition from H to Q. The deterministic transitions are indicated by solid lines in Fig. 2 We use the Iverson brackets [·] to denote an if-statement: if the condition inside the brackets evaluates to true, the cell moves from one state to the other. With this notation, we describe the deterministic transitions as

(1) $$SG2\to G1:\left[\Delta t_{s}\geq T_{SG2}\right],$$

(2) $$G1\;\to Q:\left[\Delta t_{s}\geq T_{G1}\right],$$

(3) $$Q\;\to H:\left[u_{n}<u_{n}^{H}\right],\;\text{and}$$

(4) $$H\to Q:\left[u_{n}\geq u_{n}^{H}\right].$$

To characterize the stochastic transitions, we first introduce two functions ς and ϱ [17, 15] appearing repeatedly in the transition probabilities describing a smoothed Heaviside function and linear increase, respectively. The functions are given by the following equations:

(5) $$\varsigma (x;\;a,\;b,\;\overset{‾}{x})\;=1-\exp\left(-\left(a+\frac{1}{1\;+\;\exp(2\;\cdot \;b\;\cdot \;(x\;-\;\overset{‾}{x}))}\right)\Delta t\right),\;\text{and}$$

(6) $$\varrho (x;c,\overset{‾}{x})\;=1-\exp\left(-\;max\left(c\cdot \frac{x\;-\;\overset{‾}{x}}{1\;-\;\overset{‾}{x}},0\right)\Delta t\right).$$

The dependent variable x and its parameters are separated by a semi-colon. The construction of the functions implicitly assumes bounded values $x,\;\overset{‾}{x}\in [0,\;1]\subset R$. Moreover, Δt denotes the simulation time step. For ς, the parameter a offsets the function along the y-axis, the parameter b models the sharpness of the transition, and the parameter $\overset{‾}{x}$ defines the transition point. For ϱ, the parameter a describes the slope, and $\overset{‾}{x}$ defines the starting point of the linear increase.

The stochastic transitions in the cell cycle are indicated by the dashed lines in Fig. 2. We extend the work of [15, 16, 18, 17] to account for the concentration of TRA and DOX. The transition probability for a tumor cell at position $\vec{x}$ from Q→SG2 is modeled as

(7) $$P_{Q\to SG2}=\varrho \left(u_{n}(\vec{x});c_{Q\to SG2},u_{n}^{Q\to SG2}\right)\cdot \exp(-\lambda_{Q\to SG2}u_{t}(\vec{x}))$$

where we introduce three parameters characterizing the Q→SG2 transition indicated by a sub- or superscript. Note that $\lambda_{Q\to SG2}\geq 0$. The exponential suppression is introduced because TRA leads to cell cycle arrest [58]. Introducing more parameters, we express the remaining stochastic transitions as

(8) $$P_{Q\to D}\;=\varsigma \left(u_{n}(\vec{x});a_{Q\to d},b_{Q\to d},u_{n}^{Q\to d}\right)\cdot \left(1+\xi_{d}^{Q\to D}u_{d}+\xi_{t}^{Q\to D}u_{t}+\xi_{dt}^{Q\to D}u_{d}u_{t}\right),$$

(9) $$P_{SG2\to SG2}=\varrho \left(u_{d}(\vec{x});c_{SG2\to SG2},u_{d}^{SG2\to SG2}\right),$$

(10) $$P_{SG2\to D}=\varrho \left(u_{n}(\vec{x});c_{SG2\to D},u_{n}^{SG2\to D}\right),$$

(11) $$P_{H\to D}=\left(r_{H\to D}\cdot \Delta t\right)\cdot \left(1+\xi_{d}^{H\to D}u_{d}+\xi_{t}^{H\to D}u_{t}+\xi_{dt}t^{H\to D}u_{d}u_{t}\right).$$

Here, linear and cross terms are added to parametrize the treatment effect. These terms appear in the Q→D and H→D transitions. We further introduce the SG2→SG2 transition triggering a reset of the internal clock. This transition models DOX’s ability to interfere with the DNA duplication process via intercalation [45, 46]. If the DNA duplication process fails, cells may die which we capture with the added SG2→D transition. Theoretically, the probabilities for Q→D and H→D may exceed 1 for certain parameter choices; practically however, this does not harm the implementation. One may formally rewrite the transitions as *min*(*max*(0, ●), 1) to ensure a proper probability interpretation. We note that the cell cycle is parametrized by a total of 20 parameters (see Tab. B.6 in Appendix B for all parameter values).

#### 3.1.2. Cell-cell forces

The cell-cell forces are taken from Rocha et al. [16]. In summary, the cells are represented as spheres with three radii (action, regular, and nuclear). The cell-to-cell force is a two-particle force depending on the distance d between two cells. It has adhesive and repulsive components that depend on the overlap of the different radii.

To compute the displacement of cell (i), all contributions from all other cells are summed; e.g.,

(12) $${\vec{F}}_{i}=\sum_{j\neq i}{\vec{F}}_{ij}.$$

We note that the force is zero if $d\geq R_{A}$, where $R_{A}$ is the sum of the two action radii of the cells involved in the interaction. In other words, the force is zero if cells do not overlap. Thus, we can define an index set

(13) $${𝒩}_{i}=\{j\neq i|d({\vec{x}}_{i},{\vec{x}}_{j})\leq 2\cdot \underset{k}{max}\;(r_{a}^{\left(k\right)})\}$$

containing all neighbor cells whose action radii $\left(r_{a}\right)$ overlap with the one of cell-i, and compute the force as

(14) $${\vec{F}}_{i}=\sum_{j\in {𝒩}_{i}}{\vec{F}}_{ij}.$$

This is significantly cheaper to compute because only iterates over a small index set are used rather than all other cells. Computationally, we determine the index set ${𝒩}_{j}$ through an efficient neighbor query based on an artificial, uniform grid with a discretization length $h=2\cdot {max}_{k}\;(r_{a}^{\left(k\right)})$, i.e., twice the largest action radius observable in the simulation at this time [13, 14]. After computing ${\vec{F}}_{i}$, we update the position of the cell (i) as

(15) $${\vec{x}}_{i}(t+\Delta t)={\vec{x}}_{i}(t)+\eta {\vec{F}}_{i}\cdot \Delta t\;,$$

where η is a viscosity parameter describing the linear relationship between displacement and force. We note that we do not consider forces between the tumor cells and vessels which we discuss next.

### 3.2. Blood Vessels and Angiogenesis

To model the nutrient supply, the vasculature is decomposed into small cylindrical compartments. Each compartment is computationally represented by a cylindrical agent. We base our implementation on BioDynaMo’s neurite class [13] whose equations are detailed in [59].

More broadly viewed, the vasculature is represented as a linked, tree-like data structure. The structure is realized by assigning three references to each cylindrical vessel-agent containing the address of its unique predecessor and the optional addresses of up to two successors. We refer hereafter to these as mother and daughters. If a vessel-agent has no daughter, it is called the terminal end or tip cell; if it has one daughter, it is part of a regular, longer vessel segment; and if it has two daughters, we call it a branching point.

Assuming a given, initial vasculature, the individual vessel-agents evolve independently from each other based on locally available information. In the language of agent-based modeling, each agent executes the same stochastic behaviors (rules) describing how the object changes in a time step Δt depending on the local information. In this work, we focus on VEGF-triggered sprouting angiogenesis. Our stochastic rules differentiate between tip cells, the cells embedded in a regular vessel segment, and the branching points. The latter remain unchanged because they can neither form any further branches nor can they extend into any direction.

Any vessel agent that is part of the normal vasculature with a single mother and a single daughter is a candidate for branching and, therefore, can create a new tip cell. If the agent is allowed to branch depends on three criteria. First, we require the concentration of VEGF at the agent’s position to surpass a threshold $u_{v}(\vec{x})\geq u_{v}^{\text{thres}}$. Second, it has been observed that new tip cells only form, if there are no other tip cells in its vicinity. Hence, we require a minimum Euclidean distance $d_{\text{tip}}$ to the closest tip cell (see discussions and references in [18, 42]). In other words, denoting the set of all tip cells as 𝒯, we demand that

(16) $$\underset{k\in 𝒯}{min}\;(\left\|\vec{x}-{\vec{x}}_{k}\right\|)>d_{tip}.$$

Thirdly, to ensure the mechanical stability of the vessel, branching points must be separated by a minimal distance $d_{\text{branch}}$ measured along the vessel. Denoting the curve defined by the vessel from the agent to the preceding and succeeding branching point as $\Gamma_{p}$ and $\Gamma_{s}$, respectively, a valid point for branching satisfies

(17) $$\int_{\Gamma_{p}}\;ds>d_{branch}\;\text{and}\;\int_{\Gamma_{s}}\;ds>d_{branch}.$$

To evaluate the tip cell distance criteria, we leverage an octree implementation [60] updated after each simulation time step. To evaluate the distance to the branches, we iterate over the tree structures.

If all three criteria are satisfied, the agent evaluates a stochastic branching rule: if the generated random uniform number X∼U(0,1) is smaller than the sprouting probability $p_{s}=p_{s,\text{rate}}\cdot \Delta t$, the agent creates a second daughter whose cylinder axis lies on a (random) cone around the VEGF gradient $\nabla u_{v}$. We remark that we choose the parameter $p_{s,\text{rate}}$ to be very small, e.g., it satisfies $p_{s,\text{rate}}\cdot \Delta t\ll 1$ for reasonable choices of Δt.

Tip cells, on the contrary, are never candidates for branching, i.e., we do not split vessels at the terminal end. Tip cells follow the VEGF gradient $\nabla u_{v}$ to establish the vasculature in undersupplied regions characterized by high VEGF concentrations. We allow growth if the gradient at the tip cell’s position surpasses a threshold $\nabla u_{v}(\vec{x})$.

When growing, we elongate the tip cells in the direction of the vector

(18) $$w_{1}\nabla u_{v}(\vec{x})+w_{2}\vec{a}+w_{3}X_{3},$$

where $w_{1},\;w_{2},\;w_{3}\in R^{+}$ are the modeling weights, $\vec{a}$ denotes the axis of the cylinder, and $X_{3}∼U(-1,\;1{)}^{3}$. The modeling weights determine the direction of the growth, i.e., increasing $w_{1}$ leads to vessels that follow the gradient more closely, increasing $w_{2}$ creates inert vessels barely changing their directions, and increasing $w_{3}$ allows more and more randomness in the growth.

To avoid unlimited growth after tip cell selection, a criterion to determine when to stop the growth is needed. Multiple criteria may be used; e.g., large VEGF concentrations, strong gradients, or some engineered criteria such as the quotient of the gradient magnitude and the concentration. In practice, stopping the growth once vessels reach high gradients proved effective.

The description of the initial vasculature is taken up in Section 4. To connect the new, evolving vasculature to the initial structure, we first branch from a cylinder with diameter $d_{0}$. The diameter of the new branch is computed as

(19) $$d_{1}=max(5\mu m,\;min\left(0.8\cdot d_{0},\;20\mu m\right)).$$

Furthermore, the diameter is decreased along the vessels. Typically, we elongate the cylinders until they reach a length of 10μm. We then split them into two cylinders of length 9μm and 1μm. Denoting the initial diameter as $d_{0}$, the diameter of the second agent is computed as

(20) $$d_{1}=max(5\mu m,\;min\left(0.98\cdot d_{0},\;20\mu m\right)).$$

These heuristic criteria allow us to connect the microvasculature to larger initial vessels smoothly.

Lastly, we model the effect of TRA treatment on the vessel. TRA has been shown to regularize the vasculature and improve the supply properties [54, 55]. We capture this effect by modulating the DOX supply, φ(t), with a time-dependent supply factor, χ(t),

(21) φ(t)=1+χ(t),

where a capacitor like ODE describes the time dependence

(22) $$\frac{d\chi }{dt}=\left\{\begin{array}{ll} \frac{1}{\tau_{↑}}\left(\chi_{max}-\chi \right) & \;\text{during}\;\text{TRA}\;\text{treatment,}\;\\ \frac{-1}{\tau_{↓}}\chi & \text{else.} \end{array}\right.$$

**Figure 3:**
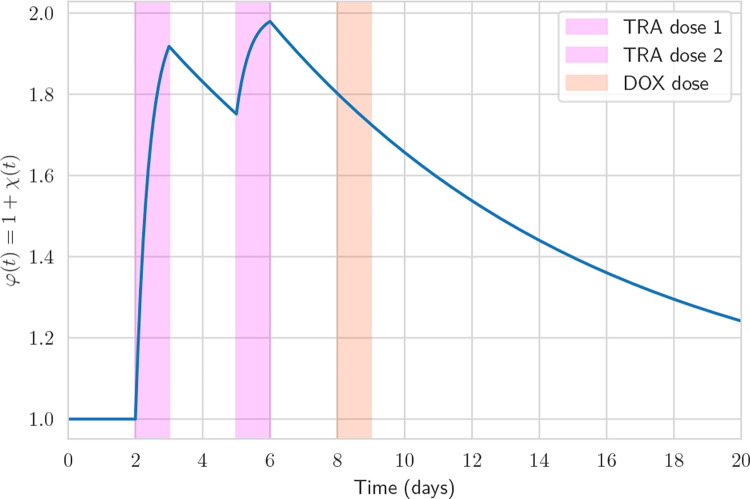
Exemplary evolution of the DOX supply φ(t) during a treatment protocol. In this illustration, one may expect roughly 75% more DOX to be delivered via the vasculature compared to no TRA treatment. Note that φ(t) remains unaffected by DOX.

An example of the time dependence of the supply factor is given in Fig. 3.

### 3.3. Continuum models

Our model involves four continua: the nutrients, VEGF, and the drugs DOX and TRA. Recall that we denote their concentration as $u_{n},\;u_{v},\;u_{d}$, and $u_{t}$, respectively. In brief, the four continua have the following roles. The nutrients model the energy supply of the system. The more nutrients, the more likely the tumor cell transition into proliferative states and multiply. In the absence of nutrients, cells become hypoxic and eventually die (necrosis). VEGF is the signaling pathway allowing dying cells to trigger angiogenesis and attract blood vessels improving the nutrient supply. TRA and DOX disturb the regular cell cycle, prohibiting proliferation and favoring transitions into hypoxic or dead states.

Assuming no chemical interactions between the different substances, each of the four substances diffuse independently in the simulation domain and may naturally decay over time. The parameters depend on the substance under consideration. The tumor cells and blood vessels act as source and sink terms in the continuum models and we denote them as $c(\vec{x};\vec{\alpha })$ and $v(\vec{x};\vec{\beta })$, respectively, where $\vec{\alpha }$ and $\vec{\beta }$ are parameter vectors. Both functions are effectively a sum of δ-distributions with strictly positive coefficients, e.g., $c(\vec{x};\vec{\alpha })=\sum_{j}\;\alpha_{j}\delta (\vec{x}-{\vec{x}}_{j})$ and $v(\vec{x};\vec{\beta })=\sum_{j}\;\beta_{j}\delta (\vec{x}-{\vec{x}}_{j})$ with $\alpha_{j},\;\beta_{j}>0$ (more details for the coupling in Section 3.4). The equations governing the continuum model are

(23) $$\left(\frac{\partial }{\partial t}-\nabla \cdot D_{n}\nabla +\lambda_{n}\right)u_{n}=-u_{n}c_{{\vec{\alpha }}_{n}}+\left(1-u_{n}\right)v_{{\vec{\beta }}_{n}}\;\;\text{with}\;\;\vec{n}\cdot D_{n}\nabla u_{n}=0\;\text{on}\;\partial \Omega ,$$

(24) $$\left(\frac{\partial }{\partial t}-\nabla \cdot D_{v}\nabla +\lambda_{v}\right)u_{v}=+\left(1-u_{v}\right)c_{{\vec{\alpha }}_{v}}-u_{v}v_{{\vec{\beta }}_{v}}\;\text{with}\;\vec{n}\cdot D_{v}\nabla u_{v}=0\;\text{on}\;\partial \Omega ,$$

(25) $$\left(\frac{\partial }{\partial t}-\nabla \cdot D_{d}\nabla +\lambda_{d}\right)u_{d}=-u_{d}c_{{\vec{\alpha }}_{d}}+\varphi (t)\left(1-u_{d}\right)v_{{\vec{\beta }}_{d}}\;\text{with}\;\vec{n}\cdot D_{d}\nabla u_{d}=0\;\text{on}\;\partial \Omega ,$$

(26) $$\left(\frac{\partial }{\partial t}-\nabla \cdot D_{t}\nabla +\lambda_{t}\right)u_{t}=-u_{t}c_{{\vec{\alpha }}_{t}}+\left(1-u_{t}\right)v_{{\vec{\beta }}_{t}}\;\text{with}\;\vec{n}\cdot D_{t}\nabla u_{t}=0\;\text{on}\;\partial \Omega .$$

Here, the diffusion and decay constants are labeled as $D_{i}$ and $\lambda_{i}$, respectively. The terms $\left(u_{i}\right)$ and $\left(1-u_{i}\right)$ ensure that the concentration remains bounded by zero and one. They also indicate whether the tumor cells and the blood vessel act as a source or a sink. For instance, tumor cells secrete VEGF but consume nutrients, DOX, and TRA. For the blood vessels, the opposite is true, i.e., they consume VEGF but provide nutrients, DOX, and TRA. The factor φ(t) was defined in Eq. 21 and 22.

The equations are solved with a finite difference scheme on a cube domain. We use the forward difference in time and the central difference in space, a scheme commonly abbreviated as FTCS scheme. Labeling the points in the (isotropic) lattice with the triple (i,j,k) and denoting the concentration at this point at time step n as $u_{i,j,k}^{n}$, the discrete stencil computation to evolve the continuum models over time is given by

(27) $$\begin{array}{l} u_{i,j,k}^{n+1}=\;(1-\lambda \Delta t)u_{i,j,k}^{n}\\ \;+\;\frac{D\Delta t}{h^{2}}\cdot \left(u_{i+1,j,k}^{n}+u_{i-1,j,k}^{n}+u_{i,j+1,k}^{n}+u_{i,j-1,k}^{n}+u_{i,j,k+1}^{n}+u_{i,j,k-1}^{n}-6u_{i,j,k}^{n}\right)\\ \;+\;\Delta t(1-u_{i,j,k}^{n})\cdot A_{+}(i,j,k)-\Delta t(u_{i,j,k}^{n})\cdot A_{-}(i,j,k), \end{array}$$

where h is the grid size, i.e., the distance between neighboring points. $A_{+}$ and $A_{-}$ characterize all agent source and sink terms, respectively. For the discrete setting, the distribution $\delta (\vec{x})$ equals one if the grid point (i,j,k) is the closest one, and is zero otherwise. In other words, if the point $\vec{y}$ is labeled as (l,m,n), the distribution $\delta (\vec{x}-\vec{y})$ reduces to a product of Kronecker deltas $\delta_{il}\delta_{jm}\delta_{kn}$. For stability, the time step Δt is bounded from above by

(28) $$\left(\lambda +\frac{12D}{h^{2}}\right)\Delta t\leq 2,$$

which follows from a standard stability analysis of the finite difference scheme.

### 3.4. Coupling of ABM and continuum

The continuum and the agent-based model are coupled: agents influence the evolution of the continuum via the source and sink terms, and the continuum values determine how the tumor cells progress in the cell cycle and drive the growth of the vasculature. For the latter, the interactions have been detailed in Section 3.1 and 3.2. For both agent types, the location of its center $\vec{x}$ is identified and the closest grid value $u_{i,j,k}$ is retrieved when we evaluate the probabilities with dependencies on the concentration of nutrients, VEGF, DOX, or TRA.

A detailed explanation of the source and sink terms is warranted. We denote the set of all tumor cells as 𝒯 and the set of all blood vessel agents as 𝒱. For the tumor cells, an agent i is identified by its center coordinate ${\vec{x}}_{i}$. The function $c(\vec{x},\vec{\alpha_{i}})(i=n,\;v,\;d,\;t)$ takes on the general form

(29) $$c\left(\vec{x},\vec{\alpha_{i}}\right)=\sum_{k\in 𝒯}\alpha_{i,k}\delta \left(\vec{x}-{\vec{x}}_{k}\right),$$

where the coefficients $\alpha_{i,k}$ are functions modeling a dependence on the agents attributes. For instance, the consumption of nutrients could be chosen to be proportional to the cell size (surface or volume). Ultimately, only the VEGF model leverages the function character of the coefficients because only hypoxic cells secrete VEGF. Thus, the coefficients read

(30) $$\alpha_{v,k}=\alpha_{v}\cdot \left\{\begin{array}{l} 0\;\text{if}\;s_{k}\neq H\\ 1\;\text{if}\;s_{k}=H \end{array}\;\;,\right.$$

with the positive, scalar parameter $\alpha_{v}$. For all other continua (i=n,d,t), we reduce the coefficients to a scalar dependence

(31) $$c\left(\vec{x},\vec{\alpha_{i}}\right)=\alpha_{i}\sum_{k\in 𝒯}\delta \left(\vec{x}-{\vec{x}}_{i}\right),$$

indicating that the source and sink terms only depend on the presence of the agent but not on any further attributes. We note that dead cells are exempt from the interactions because they neither consume nutrients nor secrete VEGF.

For the blood vessel, we reduce an agent to a number of points along the cylinder’s center line. For a vessel-agent i, the number of points $m_{i}$ is determined at each time step based on its length $l_{i}$ and the smallest grid constant $h_{j}$ (j=n,v,d,t) as

(32) $$m_{i}=max\left(3,\text{ceil}\left(\frac{2l_{i}}{{min}_{j}\;h_{j}}+1\right)\right),$$

where ceil :R→N maps a real number to the next largest integer. For convenience, we define a line-$\delta \;\left(\delta_{l}\right)$ for the vessel-agent i as

(33) $$\delta_{l,i}\left(\vec{x}\right)=\frac{2\pi r_{i}l_{i}}{m_{i}}\sum_{k=1}^{m_{i}}\delta \left(\vec{x}-{\vec{x}}_{k,m_{i}}\right),$$

where the discretization points are given by

(34) $${\vec{x}}_{k,m_{i}}={\vec{x}}_{i}^{s}+(k+1)\frac{{\vec{x}}_{i}^{e}\;-\;{\vec{x}}_{i}^{s}}{m_{i}}.$$

Here, ${\vec{x}}_{i}^{s}$ and ${\vec{x}}_{i}^{e}$ denote the start and endpoint of the cylinder’s center line. Note that the line-δ scales automatically the source and sink terms with the agent’s surface and distributes the total contribution evenly between the points. The terms are eventually given by

(35) $$v\left(\vec{x},{\vec{\beta }}_{j}\right)=\sum_{i\in 𝒱}\beta_{j,i}\cdot \delta_{l,i}(\vec{x})=\beta_{j}\sum_{i\in 𝒱}\delta_{l,i}(\vec{x}),$$

where we again simplify $\beta_{j}$ to have no dependence on the agent’s attribute. However, the coefficients $\beta_{d}$ and $\beta_{t}$ involve a time dependence; they are only non-zero during the DOX and TRA treatment. The coupling concludes our model description. While the individual modules are fairly simple, the number of modules and their interactions form a complex system parameterized by many parameters. We discuss the parameter choices and experimental setup in the next section.

## 4. Experimental Setup, Parameter Choice, and Reproducibility

In this section, we discuss the initialization of the simulation, the choice of parameters, and how to reproduce the findings of the present work. We begin by detailing the initial vasculature and tumor setup. We continue with different parameter sets. First, we discuss the parameters for the cell geometries and the forces between them. We proceed with the parameters related to the stochastic cell cycle and angiogenesis. Lastly, we discuss the continuum and coupling parameters. All parameters are given in Appendix A We conclude the section by explaining how to reproduce the computational experiments presented in Section 5 and 6.

### 4.1. Initial Simulation State

At the beginning of the simulation, the tumor cells, the vessels, and the continua are initialized to specific configurations. For the tumor cells, we randomly place cells in a sphere centered around the origin. The radius of the sphere is determined from the number of cells such that the resulting spheroid is randomly but densely packed:

(36) $$r_{spheroid}^{3}=\frac{N}{0.64}r_{cell}^{3}.$$

In application, we use data from previous work [61, 62] to define the initial structure of the vessels. The data covers a volume of roughly 1 × 1 × 2 *mm* and serves as the starting point of our angiogenesis simulation. For the continua, we enforce homogeneous Neumann boundaries and initialize the substances to a constant value (usually 0).

**Figure 4:**
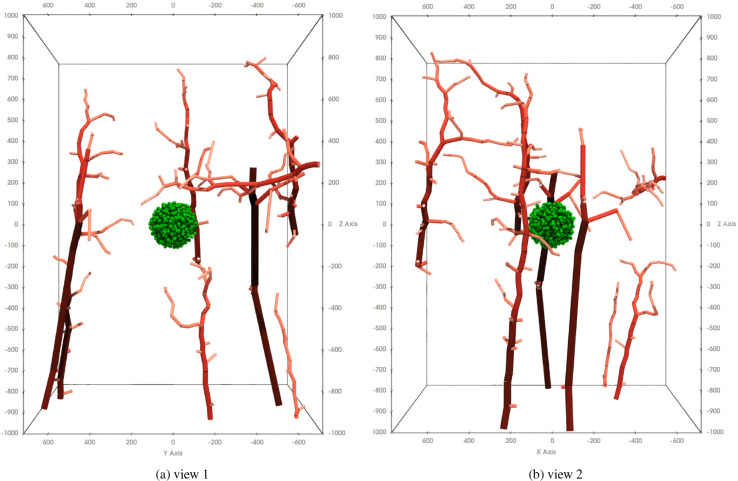
Initial configuration of the simulation. The tumor cells are shown in green independent of their state. The color of the vessel indicates the diameter; segments with larger diameters are shown in dark red, small diameters are shown in light red.

The initial state of the simulation is depicted in Fig. 4. The figure shows a tumor spheroid that is surrounded by the data-realistic vasculature. While the size of the tumor cells as well as the size and structure of the vascular are motivated by data, the relative positioning of tumor and vasculature is unspecified. Here, the tumor spheroid is placed in the empty center between the vessels; however, positioning the tumor at any other place within the simulation space is a valid choice as well.

### 4.2. Cell and Force Parameters

The tumor cells in our model describe BT-474 breast cancer cells as used in previous ABM studies [16, 18, 17, 15] and the related pre-clinical study [20]. The nuclear, regular, and action radius of the cells are chosen as 9.953μm,5.296μm, and 12.083μm, respectively [15]. See Tab. B.5. The parameters determining the strength of the repulsive and adhesive force components are taken from [17]; including the viscosity parameter found in the related source code. See Tab. B.8.

### 4.3. Cell Cycle and Angiogenesis Parameter

Lima et al. [17] calibrated their BT-474 breast cancer cell ABM with experimental data. Our cell cycle shares the deterministic and some of the stochastic transitions with their model. Here, we use their calibrated parameters as starting point. We further obtained information for effective parameters from exponential fits of Sorace’s data [20] to further guide our parameter choices. These parameters describe the effective behavior of a simple exponential surrogate, i.e., the evolution of the number of tumor cells merging the effects of all transitions in two numbers. The cell-cycle parameters are summarized in Tab. B.6.

While adopting common ideas from earlier work (tip cells following the gradient), the angiogenesis model is novel and, thus, parameters can hardly be derived from the literature. Phillips and co-workers [63] recently developed an ABM for angiogenesis and calibrated it with data from an experiment specifically designed for this scenario. They found that the sprouts extend with a speed of roughly $2\frac{\mu m}{h}$, which we adapt for this work. All other parameter choices for vessels and the angiogenesis algorithm are summarized in Tab. B.4.

### 4.4. Continuum Parameter

In this work, we consider four substances: the nutrients, VEGF, DOX, and TRA. According to Eq. (23), they obey reaction-diffusion equations, and their dynamics are determined by their respective diffusion coefficients $D_{x}$ and the decay constants $\lambda_{x}$, where x labels the substances. None of these constants are directly available.

#### 4.4.1. Diffusion Coefficients

To overcome this limitation, we first adopt Lima and coworkers’ [17] estimate for the value of the nutrient diffusion $D_{n}=50\frac{\mu m^{2}}{h}$. Intuitively, diffusion is a passive transport phenomenon and larger, heavier objects should diffuse more slowly. Einstein [64] showed that the diffusion coefficient is inversely proportional to the radius of the diffusing particles, i.e., $D∼r^{-1}$. Since it is non-trivial and beyond the scope of this work to define an effective radius for complex protein structures, we work with the simple hypothesis that the mass of a molecule or a protein structure scales cubically with the radius, e.g., $m∼r^{3}$. We conclude that $D∼m^{-1/3}$. Thus, if the masses of two particles are related by the relationship $m_{2}=\alpha m_{1}$, the respective diffusion coefficients behave as $D_{2}=\alpha^{-1/3}D_{1}$. The masses of all four diffusing molecules are known and, together with Lima’s estimate for $D_{n}$, we compute estimates for the remaining diffusion constants. The masses, values for α, and the diffusion constants are given in Tab. (1).

#### 4.4.2. Decay, Source, and Sink Terms

For the continua, the decay parameter and the tumor cell sink terms both lead to an exponential decay. Ignoring the diffusion and sink terms, the update rule for the substance concentration at a given position reads

(37) $$u^{n+1}=\left(1-\lambda^{\prime }\cdot dt\right)u^{n}.$$

If we add the sink terms of N tumor cells, we obtain the relationship

(38) $$u^{n+1}=\left(1-\lambda \cdot dt-\left(\sum_{i=1}^{N}{\bar{r}}_{i}\right)dt\right)u^{n}=\left(1-\lambda \cdot dt-\left(\sum_{i=1}^{N}\frac{r_{i}}{dx\;\cdot \;dy\;\cdot \;dz}\right)dt\right)u^{n},$$

where we rewrite the consumed concentration $\overset{‾}{r}$ in terms of the consumed amount r. Acknowledging that $r_{i}$ is independent of the tumor cell under consideration, we use a homogenization approach to rewrite the previous equation in terms of the tumor cell density ρ:

(39) $$u^{n+1}=\left(1-\lambda \cdot dt-\frac{rN}{dx\;\cdot \;dy\;\cdot \;dz}dt\right)u^{n}=(1-\lambda \cdot dt-r\cdot \rho (x,y,z)dt)u^{n}.$$

Comparing the previous equations yields

(40) $$\lambda^{\prime }=\lambda +N\overset{‾}{r}=\lambda +r\rho (x,y,z),$$

displaying that our model effectively shows a global decay of the substances as well as a local decay depending on the density distribution of the tumor cells. Furthermore, 40 allows us to compare the parameters λ and r with data and simple exponential surrogates $\lambda^{\prime }$ attributing effects to tumor cell presence or not.

DOX engages in different chemical reactions whose effect we model with the decay constant and the sink terms. According to the FDA [65], DOX shows a terminal half-life of 20 to 48 hours. Data for TRA suggest a dose-dependent half-life of 1.7 to 28 days [66, 67]. These data imply the decay constants of $\lambda_{d}^{\prime }\in \left[14.4\cdot 10^{-3},\;34.7\cdot 10^{-3}\right]h^{-1}$ and $\lambda_{t}^{\prime }\in \left[1.0\cdot 10^{-3},\;17.0\cdot 10^{-3}\right]h^{-1}$, respectively. However, the data stems from generic experiments and is not necessarily representative. Lima and coworkers [57] recently calibrated their ODE model to fit the same data that we consider here and obtained the decay constants $\lambda_{b}^{\prime }=12.0\cdot 10^{-3}h^{-1}$ and $\lambda_{t}^{\prime }=20.0\cdot 10^{-3}h^{-1}$. For DOX, their estimates are slightly below the data range, and for TRA slightly above.

We realize that DOX is unspecific in its nature while TRA only interacts with the HER2 receptor of the tumor cells. We further consider the initial vasculature and the not explicitly modeled regular tissue to be in equilibrium. Consequently, the excess nutrients supplied via the new vasculature should primarily be consumed via the tumor cells (see also the Warburg effect [68, 69]). Using these findings and further values from [17], we summarize all our parameter choices regarding the continua in Tab. B.9 and B.7.

### 4.5. Reproducibility

To ensure transparency and reproducibility, we share all source code and data used in the project^2^. To reproduce the results of the following sections (i.e., Section 5and 6), we need to fix four key components:

- the version of the BioDynaMo source code,
- the version of the application source code including all possible changes,
- the parameters used for the simulation, and
- the postprocessing pipeline.

We share two repositories on GitHub: (1) the repository TobiasDuswald/angiogenesis contains the application code, and (2) the repository TobiasDuswald/bdm-angiogenesis-reproducer contains the parameters, possible patches to the source code, and postprocessing routines. We structured the latter repository such that it contains one folder in “experiments/*” for each result shown in the main text. The respective folders have the information to adequately initialize the code and parameters for the simulation runs. For convenience, the initialization, build process, execution, and post-processing of each computational experiment is wrapped in bash scripts. Thus, to reproduce any figure in the main text, the reader only needs to run a single bash script. Note, however, that BioDynaMo simulations are not bit-reproducible at the time of writing.

## 5. Results

In this section, we present the results of our computational experiments. They are ordered such that their complexity gradually increases. We begin with simulating the growth of tumor spheroids in the absence of any vasculature and treatment in Section 5.1. Next, we investigate different vascular patterns arising from the angiogenesis algorithm in Section 5.2. Afterwards, we demonstrate the fully coupled model by simulating the vascular growth and treatment. We first establish a clear and visual understanding of the different phases of the simulation by showing a conceptual simulation in Section 5.3. In Section 5.4, we then focus on the key quantity of interest, the tumor volume, and show how it evolves over time for different treatment scenarios. We qualitatively compare these results to Sorace and co-workers’ observations [20, 57]. For all simulations involving the vasculature, we consider the initial setup described in the previous section and summarized in Fig. 4.

### 5.1. Tumor Spheroids and the Hypoxic Threshold

In this subsection, we consider the growth of a tumor spheroid ignoring vasculature and treatment protocols $\left(u_{v}=u_{d}=u_{t}=0\right)$. To initialize the simulation, we set the nutrients to a constant value and employ Dirichlet boundaries with the same, constant value; i.e. $u_{n}(t=0)=0.5$ and ${\left.u_{n}(t)\right|}_{\partial \Omega }=0.5$. In the absence of vasculature, the Dirichlet boundary conditions effectively act as nutrient supply. Here, we restrict ourselves to investigating the hypoxic threshold $u_{n}^{H}$ because earlier 2D studies showed that this parameter has significant influence on the number of proliferative cell driving the tumor growth [17, Fig. 6B]. The parameter marks the transition from the quiescent to the hypoxic state and, thus, prohibits cell proliferation in regions with insufficient nutrient availability $\left(u_{n}<\sigma_{n}^{H}\right)$. In other words, one may say that the hypoxic threshold defines hypoxic and proliferative regions via a level-set function on $u_{n}$. For this experiment, we place 500 tumor cells in the center of our cubical simulation domain and simulate the growth of the tumor for 200 days with a time step of one minute for different hypoxic thresholds $u_{n}^{H}\in \{0.15,\;0.13,\;0.12,\;0.11\}$.

**Figure 5:**
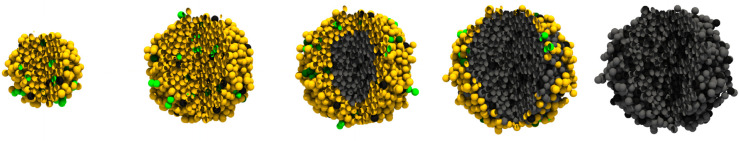
Evolution of the tumor spheroid for $u_{n}^{H}=0.15$. Time progresses from left to right. The colors indicate the state: yellow (Q), bright green (G1), dark green (SG2), gray (H), and black (D).

Figure 5 shows the spheroid over time for the largest hypoxic threshold $u_{n}^{H}=0.15$. The cells are initially in proliferative states, consume nutrients, and the spheroid grows. Over time, a hypoxic region in the center of the spheroid begins to form. The border between hypoxic and proliferative regions is implicitly visualized as the sharp transition zone between proliferative (yellow, green) and hypoxic (grey, dark blue) tumor cells. The time-dependent border may be defined as a hypersurface satisfying $u_{n}(x,t)=u_{n}^{H}$. Its dynamics together with the spacial structure of the tumor effectively determine the evolution of the spheroid. The more tumor cells lie outside the hypersurface, the more cells participate in the exponential cell proliferation. Parameters such es the nutrient consumption and the hypoxic threshold define the shape and dynamics of this surface. The dynamics of the tumor growth effectively comes down to a race between the spatial tumor extend and the hypersurface, i.e., if the hypersurface can spread quicker than the tumor to eventually enclose the entire spheroid and stop the growth. This is the case for $u_{n}^{H}=0.15$ where the tumor eventually stops growing and dies off, see Fig. 5 on the right.

**Figure 6:**
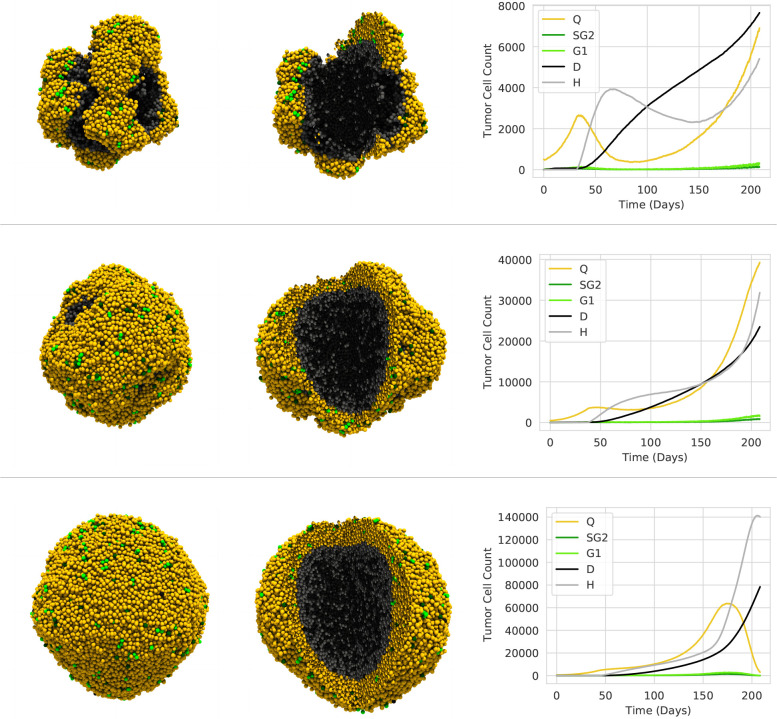
Evolution of tumor spheroids. The rows correspond to different hypoxic threshold $u_{n}^{H}\in \{0.13,\;0.12,\;0.11\}$ (top to bottom). The first columns shows all cells, the second colum adds a cut-out revealing the inner structure, and the last column shows the number of cells in different states over time. The spheroids for the hypoxic thresholds 0.13,0.12, and 0.11 are shown after different simulation times, i.e., after 200,165, and 145 days, respectively. The boundaries start to affect the simulation afterwards. The colors indicate the state: yellow (Q), bright green (G1), dark green (SG2), gray (H), and dark blue (D).

With decreasing $u_{n}^{H}$, we observe vastly different growth patterns in Fig. 6. We display the spheroids for the remaining hypoxic thresholds $\left(u_{n}^{H}\in \{0.13,\;0.12,\;0.11\}\right)$ at specific time points in the simulation (i.e., 200, 165, and 145 days). We further show the dynamics with a graph of the cell numbers in different states over time. In Fig. 6, each row corresponds to a hypoxic threshold. In contrast to Fig. 5, the hypersurface cannot cover the entire spheroid and the growth never stops completely. For instance for $u_{n}^{H}=0.13$ (Fig. 6 top row), almost all cells transition into the hypoxic state after roughly 50 days. However, some cells on the surface still lie outside the hypoxic regions and continue to proliferate (similar to the fourth spheroid in Fig. 5). These few cells eventually move on to form satellite tumors on the surface of the original spheroid. Further lowering the threshold to $u_{n}^{H}=0.12$ (Fig. 6, middle row), more and more cells on the surface remain in the proliferative states and larger parts of the spheroids are covered with proliferative cell populations. Once we reach $u_{n}^{H}=0.12$ (Fig. 6, bottom row), the entire surface remains proliferative throughout the simulation and the tumor shows the characteristic proliferative hull around a necrotic core. Over all, the graphs clearly show that a lower hypoxic threshold leads to stronger proliferation. It is interesting to note that the stochasticity of the system breaks its symmetry for $u_{n}^{H}\in \{0.13,\;0.12\}$. After investigating the dynamics of the tumor growth, we now shift our attention to the development of the vasculature via sprouting angiogenesis.

### 5.2. Angiogenesis

To demonstrate the process of angiogenesis, we simulate the system for an extended period of time (i.e., 14 days) and observe the resulting vasculature under the influence of different parameters. The majority of them have straight-forward interpretations. The tip cell distance $d_{tip}$, branching distance $d_{branch}$, and sprouting probabilities influence the sparsity pattern of the network. The weights combining the gradient information, randomness, and previous growth direction allow us to interpolate between a random walk and smooth curves along the gradient. The VEGF and gradient thresholds put hard limits on the signal strength that a vessel agent has to sense to form a sprout. Here, we want to focus on the parameter that is easily overlooked.

**Figure 7:**
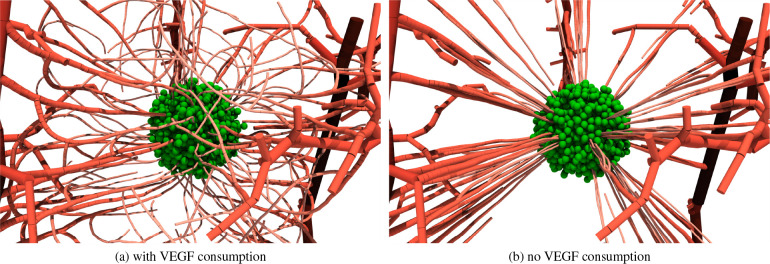
Simulated vasculature (a) with and (b) without vessels acting as sink terms. The tumor cells are shown in green independent of their state. The color of the vessel indicates the diameter; segments with larger diameters are shown in dark red, small diameters are shown in light red.

The term coupling the vessels to the VEGF concentration is the most influential parameter on the structure of the vasculature. While this effect is hard to quantify, we depict the final vasculature of the simulation with and without the coupling term in Fig. 7 (all other parameters are kept identical). It is evident that the vasculature differs significantly for the two scenarios. Without the coupling, the vessels simply grow towards the tumor center in relatively straight lines. We also note that even if the vessels branch, they follow almost the same path. In contrast, the coupling term removes VEGF from the vessel’s vicinity and, thus, it locally changes the gradient field encouraging new sprouts to grow away from its parent vessel, avoid other vessels growing towards the tumor, and search for alternative paths to revive the hypoxic regions. The network of the coupled simulation produces a significantly more diffuse network in which vessels surround the tumor rather than growing towards its center. In experimental work [70], researchers showed that typically the blood flows on the outskirts of the tumor spheroid indicating that the tumor-surrounding, diffuse network is more realistic than the one ignoring the coupling. It is worth noting that the diffuse growth process also produces vessels that, over time, grow in other VEGF rich regions other then the tumor core. Our model does not prune such growth; however, pruning mechanisms such as suggested in 62 are beyond the scope of the present work.

### 5.3. Vascular Tumor Growth and Treatment

Combining the previous sections, we now aim to simulate vascular tumor growth and treatment. We initialize the simulation with 1000 tumor cells in the center of the vasculature, see Fig. 4. We start the simulation with no nutrients, i.e., all cells take the hypoxic state and secrete VEGF. We choose to couple the vessels to the VEGF field to achieve a more realistic, tumor-surrounding micro-vasculature. The vasculature develops around the tumor over time and we subsequently expect the tumor to grow into the surrounding, nutrient-rich regions. At day 70, we turn off the vessel growth algorithm to avoid growth resulting from sprouts that may not hit the stopping criteria. We let the tumor proliferate until day 102 on which we trigger the treatment. We simulate the treatment by turning on the TRA and DOX source terms between days 102–104 and 106–108, respectively. We remark that the time scales are somewhat arbitrary; we choose them such that the tumor covers the newly vascularized region before the treatment begins.

**Figure 8:**
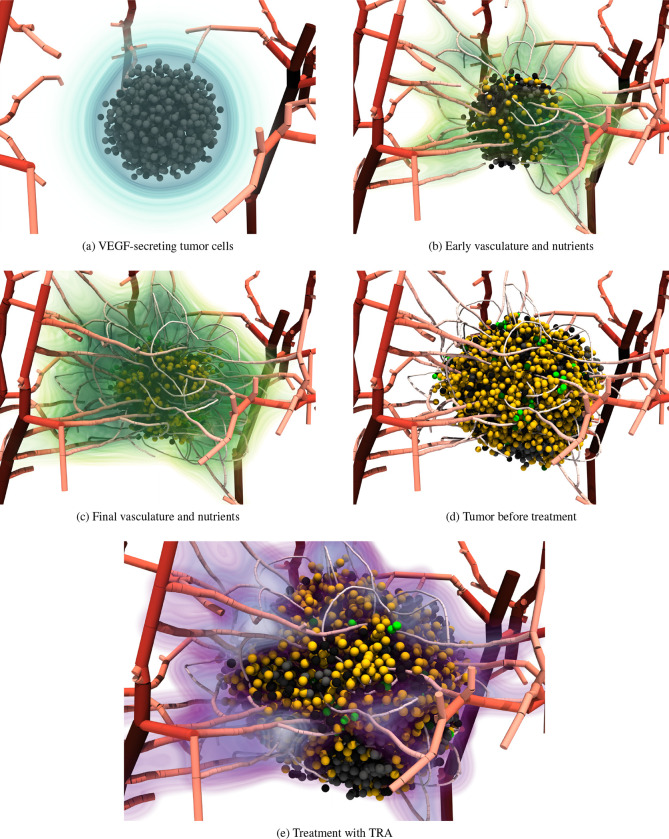
Visualization of a full simulation run with tumor cells colored by their cell state: Q (yellow), SG2 (dark green), G1 (light green), H (gray), and D (black). (a) Initial hypoxic population secreting VEGF (blue) to trigger angiogenesis. (b) First vessels reach the tumor surface and supply nutrients (green) leading to cells taking proliferative states on the surface. (c) Final state of the vasculature, i.e., we deactivated the vessel growth algorithm at this point. (d) Final tumor before treatment initialization. (e) Early stage of the treatment (TRA in purple)

In Fig. 8 we show the evolution of the simulation over time. While we start with only 1000 tumor cells, the number increases by a factor of 5 until the treatment begins. Figure 8 (a) shows the simulation briefly after the initialization. All tumor cells are in the hypoxic state and secrete VEGF displayed in blue. The dynamics of VEGF are encapsulated in the PDE (Eq. (24)) and, consequently, it diffuses from the tumor into the surrounding regions. After some time that primarily depends on the secreted amount and the diffusion coefficient, VEGF reaches the vasculature and, in reaction, sprouts form and move along the gradient towards the tumor in the center. The new vasculature supplies nutrients, and once the nutrients and vessels reach the spheroid, cells on the surface begin transitioning into proliferative states (Fig. 8(b)). Afterward, the vasculature keeps developing until terminated at day 70 (Fig. 8 (c)). The tumor evolves in the final vasculature with its proliferative ring until it reaches its largest size in Fig. 8 (d).

It is interesting to note that the model favors growth along the vessels because these regions provide more nutrients and the cells are more likely to transition into the proliferative state SG2. Thus, more tumor forms in the well-vascularized regions. Admittedly, this is not immediately evident in Fig. 8, however, the bottom region in (d) is poorly vascularized and one can see that the hypoxic cells reach all the way to the surface of the tumor spheroid (d/e). Furthermore, considering (b), we observe a fairly dense network on the right side of the tumor resulting in an outgrowth to the right in (c). Overall, the model shows expected characteristics and significant similarities to images of 3D *in vitro* studies [71].

After the tumor has formed, we trigger the treatment with TRA and DOX. Figure 8 (e) shows the tumor and the concentration of TRA (purple low, blue high) created by the vessel source terms. After the treatment, tumor cells stop proliferation and begin transitioning into the dead state inhibiting further tumor growth. The effects of the treatment are investigated in the following section.

### 5.4. Treatment Comparison

**Figure 9:**
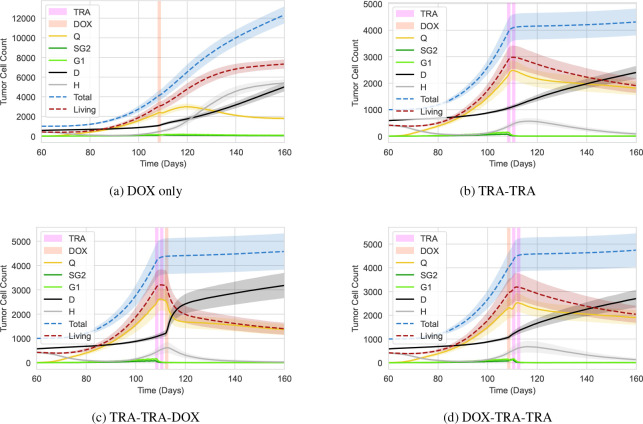
Evolution of the number of tumor cells in the different states for different treatment protocols. The line of the living cells add up all states but the dead cells. The line of the *total* number of cells further includes the dead cells.

Recall that a principal goal of this investigation is to build a model to better understand the combination of DOX and TRA for breast cancer treatment. In this section, we initialize the simulation as in the previous two experiments and Fig. 8 depicts the different simulation stages. Here, we selected the treatment parameters such that our model accurately describes Jain’s hypothesis [19] and matches the trends in the data [20]. For the treatment, we allocate three slots between the days 108–109, 110–111, and 112–112. During these intervals, the vasculature acts as source terms for the cancer drugs. We consider four treatment scenarios relating to the treatment groups 2, 3, 4, and 5 of Fig. 1, i.e., DOX only, TRA only, TRA followed by DOX, and DOX followed by TRA. For each treatment scenario, we run 10 simulation runs to account for the inherent stochasticity and plot the mean and standard deviation of the number of cells in different states over time. The results are depicted in Fig.9. All simulations use the identical set of parameters and only differ in the treatment schedule.

Figure 9 (a) shows the treatment effects when we only apply DOX. In our simulations, this protocol is ineffective and the tumor growth is barely disturbed. After the application, the quiescent cells show a slight decline but they quickly recover. Recall that DOX has a short half-life and, thus, long-term changes are not readily observed. The unaffected tumor growth agrees qualitatively with the data in Fig. 1(b).

TRA has a significantly longer half-life and we expect to see long-term effects. In Fig. 1 (c), the data shows that the TRA treatment stalls the tumor growth. Our simulations show the same pattern, e.g., in Fig. 9 (b), the tumor stops growing. This effect is modeled with the Q→SG2 suppression through TRA.

Next, we consider the scenario in which we first apply TRA and subsequently supply DOX - the test case for Jain’s hypothesis. In Fig. 9 (c), right after DOX is applied, we observe a sharp decline in quiescent cells and a strong increase in the number of dead cells. Here, the dose is significantly more effective than if only DOX is applied. This is due to the improved supply properties of the vasculature caused by the preceding TRA treatment allowing more DOX to enter the system. Among all the simulated scenarios, the TRA-TRA-DOX treatment shows the strongest treatment effect, i.e., the number of (living) tumor cells at the end is the lowest. This result agrees with Fig. 1 (e) and Jain’s hypothesis.

Lastly, we want to consider the inverse case, e.g., DOX treatment followed by TRA (Fig. 9 (d)). Our simulation results are, in this case, hard to distinguish from the case in which we solely use TRA. Unfortunately, they disagree with the data (Fig. 1 (d)) which suggests that the first dose of DOX prohibits TRA from being effective. The present model does not capture this feature. Terms involving both drugs cannot explain the observation because they do not differentiate between the order in which drugs arrive. We hypothesize that DOX either affects the vessels and therefore the supply, or effects of DOX in the cell’s internals disturb the pathways used by TRA. Both effects have not been considered in the model. While there are some hints that DOX may in fact damage the microvasculature [72], this would also harm the nutrient supply and contradict the strong growth in Fig. 11(d). Thus, we lean towards the latter and hypothesize that DOX negatively influences the way TRA can work inside the cells.

## 6. Towards Large Scale Simulations

Criticism of some ABMs has arisen because of their high computational costs and lack of scalability. Typical ABM simulations focus on small-scale systems and cannot simulate medically relevant sizes. However, recent advances in ABM software [13, 14] address many of the computational bottlenecks and provide the foundation for scaling up simulations. Leveraging these optimizations through the BioDynaMo API, we here demonstrate that our model and the associated C++ code can handle medically relevant system sizes by reproducing the pre-treatment data of Sorace’s pre-clinical study [20].

We choose a 9 × 9 × 9*mm* simulation volume. For lack of data availability, we stochastically mimic the vascular density across the simulation volume. For details, consider Appendix C. In the pre-clinical study [20], the researchers injected 10 million tumor cells into rodents. To agree with the average tumor volume observed on day seven, we initialize our simulation with 6 million tumor cells in a spheroid. We focus on the pretreatment stage and simulate from day 7 to 34 with a timestep of 10 minutes. In other words, we simulate 27 days with 3888 time steps. We discretize the continua with 22.5×22.5×22.5μm voxels (roughly the size of a tumor cell).

**Figure 10:**
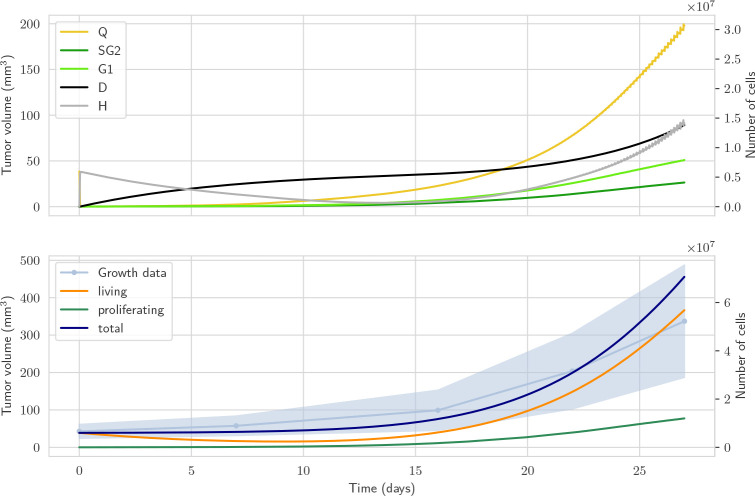
Large scale simulation: tumor volume and cell count over time. Top: volume and number of cells according to the five cell states. Bottom: Aggregated numbers proliferating (SG2+G1), living (SG2+G1+Q+H), and total (all). Tumor volume computed as $V_{tumor}=N\cdot V_{cell}/0.64$, with the measured number of cells N and a correction factor accounting for sphere packing. The displayed growth data combines the pre-treatment stage of all treatment groups in Fig. 1.

Figure 10 shows the tumor volume over time. In the beginning, all cells are in a hypoxic state. They then begin to stochastically transition into the dead state and no longer consume any nutrients. At the same time, the vasculature starts growing, and the available nutrients increase. From day three, we observe cells transitioning into proliferative states, and the tumor grows in some regions. Between days 10 and 15, we observe that almost as many cells are in the necrotic state as we initialized, indicating that most of the spheroid died, and the initial spheroid now forms a necrotic core. From day ten on, parts of the tumor are well supplied with nutrients after attracting the vasculature via VEGF, and we observe exponential growth. Between days 15 and 20, the number of hypoxic cells increases again. This trend suggests that the exponential growth of the tumor mass depleted newly vascularized regions, and parts of the tumor begin to die. The overall tumor dynamics seem reasonable and agree with the pre-treatment data (Tab A.2) as can be seen in the lower part of Fig. 10.

While starting with 6 million tumor cells and roughly 4.5 million vessel segments, the simulation concludes with 70.6 million tumor cells and 21 million vessel agents, respectively. The vessel volume, and therefore the vascular density increased by a factor of roughly 5. Overall, the simulation took approximately 7.9 days on a 72-core server with 1 TB of RAM and hyper-threading (4 x Intel(R) Xeon(R) E7–8890 v3 clocking at 2.50 GHz with four NUMA domains) and the memory usage peaked around 50 GB. It appears that the force computation is responsible for most of the computation. We expect that the runtime can still be significantly reduced by optimizing the model code.

## 7. Discussion

The computational and mathematical models and algorithms described in this work appear to be capable of simulating very complex growth of vascular structures and variations in tumor volume in environments in which drug protocols are designed and orchestrated to control, minimize, or eliminate tumor growth.

A limitation of the model is that the healthy tissue surrounding the tumor is not considered. As long as the *in silico* tumor floats in a vacuum, it is difficult to mimic the physical traits [24] such as stress, pressure, and stiffness. Effects, such as vasculature being damaged by forces, can hardly be modelled when the cells can escape into the empty space. Ignoring the forces between tumor cells and the vasculature is another limiting factor linked to the previous point. Without the healthy tissue surrounding the vasculature and tumor, the vasculature would be pushed away rather than forming a supply network. Future work should also model the cell death in more detail to free space for the proliferative tumor mass and the healthy tissue (see [73]). Moreover, our model neglects cell migration which has proven to have a significant impact on the tumor dynamics in theoretical studies [74, 75].

While the generated vascular networks appear to be realistic and organic, the growth does not entirely stop and vessels can begin growing in unexpected directions. In fact, vasculature grows less structured in the presence of a tumor; however, our model lacks pruning mechanisms for the vessels growing in random directions. Extending the model with a flow simulation seems a promising direction for further research and would give additional information based on which one may prune and optimize the vasculature (see, e.g., [62]).

Moving away from the vasculature, we note that the model has many parameters, including some that have not been properly calibrated with data. The number of parameters is, however, a by-product of the complexity that was targeted in this work. Adding more mechanisms to the model inevitably adds more parameters. Nonetheless, by building on similar models and their (partially calibrated) parameters [15, 16, 17, 18], we were able to find reasonable parameter choices for the model which we demonstrated through the model’s ability to simulate vascular tumor growth and treatment. We were able to reproduce the qualitative reaction of HER2+ breast cancer to the combination treatment with DOX and TRA. The parameter choices and modeling approach is further justified by the fact that the model produces realistic tumor volumes in agreement with the pre-clinical study [20]. However, our model ignores effects on the tissue scale (e.g., cell death and migration) and is therefore not yet adequate for predicting quantities of interest at this scale.

The models described in this work offer many interesting opportunities. They have great potential for describing small *in vitro* experiments (see, for instance, [71]). Such data would further help calibrate our model. For these small in vitro scales, parameters may be inferred using Bayesian frameworks because the model runs fast and shared-memory parallel such that frequent model evaluation may be possible. However, even though we emphasized computational efficiency during the development, the calibration of stochastic models remains a challenging subject because the repeated evaluation of the forward model may amount to substantial run times. Recent advances in Bayesian computation [76] suggest the construction of surrogates based on Gaussian processes to reduce the number of forward simulations. The method has proven efficient for other stochastic cancer models and may help to calibrate ours. Once those parameters have been calibrated, one may run large-scale simulations and compare the results to macroscopic data, as demonstrated in this work. The model and its implementation should enable researchers to bridge scales, i.e., hypothesize phenomena on microscopic, cellular scales and compare the simulation results to macroscopic data.

## 8. Conclusion

In this work, a complex hybrid model is presented together with a performant C++ implementation. The model is shown to capture many characteristics of vascular tumor growth and, qualitatively, describes the treatment effects of Doxorubicin and Trastuzumab on HER2+ breast cancer cells. Furthermore, the model and code can scale to tissue-relevant sizes and may therefore help future research to bridge scales, i.e, hypothesize cellular effects and test how they affect macroscopic quantities.

## Figures and Tables

**Table 1:** The table shows 1) the molecular mass of the diffusing molecules and proteins, 2) the factor *α* expressing the mass in terms of the glucose mass, and 3) the scale factor $\alpha^{-1/3}$ for the diffusion coefficients.

	$\text{molecular}\;\text{mass}\;[\frac{g}{mol}]$	α	$\alpha^{1/3}$	$D_{x}\;\text{in}\;[\frac{\mu m^{2}}{h}\;]$

Glucose (nutrients)	180	1	1	50.0
VEGF (monomer)	19.3 · 10^3^	107	0.21	10.5
VEGF (dimer)	38.6 · 10^3^	214	0.16	8.0
DOX	543	3	0.69	42.5
TRA	145 · 10^3^	806	0.11	5.5

**Figure C.11:**
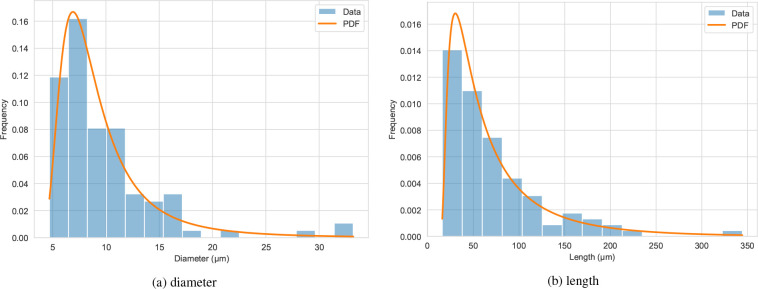
Histograms of the data [78] (blue) together with the best fitting PDF (orange). The PDF in (a) is a generalized extreme value distribution, and in (b) it is a Wald distribution.

## Footnotes

1 Available after final publication on GitHub: TobiasDuswald/angiogenesis, TobiasDuswald/bdm-angiogenesis-reproducer Currently upon request.

2 available after final publication, currently upon request

## Appendix A. Data

The data in [57, Tab. 1 and 5] contains six groups for the tumor growth before treatment begin. Generally, two sets $S_{i}=\left\{x_{1},\ldots ,x_{n_{i}}\right\}$ of real numbers with their respective cardinality $\left(n_{1},{\;n}_{2}\right)$, mean $\left(m_{1},m_{2}\right)$, and standard deviation $\left(\sigma_{1},\sigma_{2}\right)$ can be combined in one set S with its characteristics given by

(A.1)
$$n=n_{1}+n_{2},$$

(A.2)
$$m=\frac{n_{1}m_{1}\;+\;n_{2}m_{2}}{n_{1}\;+\;n_{2}},\;\text{and}$$

(A.3)
$$\sigma =\sqrt{\frac{\left(n_{1}\;-\;1\right)\sigma_{1}^{2}\;+\;\left(n_{1}\;-\;1\right)\sigma_{1}^{2}\;+\;\frac{n_{1}n_{2}}{n_{1}+n_{2}}{\left(m_{1}\;-\;m_{2}\right)}^{2}}{n_{1}\;+\;n_{2}\;-\;1}}.$$

We apply these formulas iteratively to the dataset in [57, Tab. 5] and obtain a dataset characterizing the tumor growth before treatment in a statistically more sound way. The dataset is given in Tab. A.2.

**Table A.2:** Tumor volume in cubic millimeters over time until treatment begin. Data merged from [57, Tab. 5].

Days	Combined

7	42.721 ± 18.69
14	57.53 ± 26.19
23	99.06 ± 53.76
29	203.95 ± 101.30
34	337.21 ± 150.22

## Appendix B. Model Parameter

**Table B.3:** Overview of the parameters affecting the simulation. (regular simulation, large scale)

Parameter	Notation	Value	Unit	Misc

number of tumor cells	*N*	10^3^, 6 · 10^6^	1	cube domain
lower bound	*x**_min_*, *y_min_*, *z_min_*	−1.3, −4.5	*mm*	cube domain
upper bound	*x**_max_*, *y_max_*, *z_max_*	1.3, 4.5	*mm*	cube domain
resolution continua	*N_n_, N_v_, N_d_, N_t_*	200, 400	1	voxel per dim

**Table B.4:** Overview of the parameters affecting the vessel.

Parameter	Notation	Value	Unit	Source

Bifurcation distance	*d*_*b*_	80	*μm*	−
Tip-cell distance	*d*_*t*_	150	*μm*	−
Sprouting rate	*P_s_*	0.001	1/*min*	−
Growth weight random	*W_r_*	0.2	−	−
Growth weight old	*W_o_*	0.5	−	−
Growth weight gradient	$w_{\nabla }$	0.3	−	−
Growth speed	*s*	0.033	*μm/min*	[63]
Supply increase	$\tau_{↑}$	0.4	days	−
Supply decrease	$\tau_{↓}$	10	days	−
Supply maximum	*X*_max_	9	days	−

**Table B.5:** Overview of the parameters affecting the tumor cell.

Parameter	Notation	Value	Unit	Source

cell radius	*r*_*c,u*_	9.953	*μm*	[15, 77]
nuclear cell radius	*r*_*c,n*_	5.296	*μm*	[15]
action cell radius	*r_c,a_*	12.083	*μm*	[15]

**Table B.6:** Overview of the parameters affecting the cell cycle.

Transition	Parameter	Notation	Value	Unit	Source

*SG2 → G1*	duration cell cycle	$\tau_{p}=\tau_{G1}+\tau_{SG2}$	18	hour	[15]

*G1 → Q*	duration growth phase	$\tau_{G1}$	9	hour	[15]

*D →* gone	duration apoptosis	$\tau_{D}$	8.6	hour	[15]

*Q → H*	nutrient threshold	$u_{n}^{Q\to H}$	0.09	−	

*Q → D*	nutrient threshold	$u_{n}^{Q\to D}$	0.000538	−	−
	nutrient gamma	$\gamma_{n}^{Q\to D}$	0.000408	−	−
	nutrient alpha	$\alpha_{n}^{Q\to D}$	6.8. 10^−6^	−	−
	nutrient k	$k_{n}^{Q\to D}$	50.0	−	−
	DOX zeta	$\zeta_{d}^{Q\to D}$	100	−	−
	TRA zeta	$\zeta_{t}^{Q\to D}$	0	−	−

	Cross term	$\zeta_{dt}^{Q\to D}$	0	−	−

*Q → SG2*	nutrient threshold	$u_{n}^{Q\to SG2}$	0.0538	−	[17]
	nutrient alpha	$\alpha_{n}^{Q\to SG2}$	0.000821	−	[17]
	decay with TRA	$a_{t}^{Q\to SG2}$	30	−	−

*SG2 → SG2*	DOX threshold	$u_{d}^{SG2\to SG2}$	0.001	−	−
	DOX alpha	$\alpha_{d}^{SG\to SG2}$	0.1	−	−

*SG2 → D*	DOX threshold	$u_{d}^{SG2\to D}$	0.001	−	−
	DOX alpha	$\alpha_{d}^{SG\to D}$	0.001	−	−

*H → D*	Base rate	*r*	0.00001	−	−
	DOX zeta	$\zeta_{d}^{H\to D}$	100	−	−
	TRA zeta	$\zeta_{t}^{H\to D}$	0	−	−
	Cross term	$\zeta_{dt}^{H\to D}$	0	−	−

**Table B.7:** Overview of the parameters affecting the agent - continuum interaction. Estimation explained in the main text. Note that *c*_*v*_ and *v*_*v*_ may be chosen significantly smaller without affecting the results but we report the parameters used for the numerical experiments.

Agent	Parameter	Notation	Value	Unit	Source

Tumor Cell	nutrient consumption	*c*_*n*_	0.0483	*h* ^−1^	[17]
Tumor Cell	VEGF supply	*c*_*v*_	2.73	*h* ^−1^	−
Tumor Cell	DOX consumption	*c_d_*	0.0	*h* ^−1^	−
Tumor Cell	TRA consumption	*c_t_*	0.000983	*h* ^−1^	−

Vessel	Nutrient supply	*V_n_*	0.0000819	1 /(*μm*^*2*^ · *h*)	−
Vessel	VEGF consumption	*V_v_*	0.109239	1 /(*μm*^*2*^ · *h*)	−
Vessel	DOX supply	*V_d_*	0.000136	1 /(*μm*^*2*^ · *h*)	−
Vessel	TRA supply	*V_t_*	0.002730	1 / (*μm*^*2*^ · *h*)	−

Vessel	VEGF threshold	$v_{v}^{sprout}$	0.001	1	−
Vessel	VEGF gradient threshold	$v_{\nabla v}^{sprout}$	0.00001	1	−

**Table B.8:** Overview of the parameters affecting the forces.

Parameter	Notation	Value	Unit	Source

Cell-cell adhesion	*f_a_*	0.0489	*μm/min*	[17]
Cell-cell repulsion	*f_r_*	10.0	*μm/min*	[17]
Maximal speed	*v*_*max*_	10.0	*μm/min*	[17]
Viscosity	*v*	2.0	−	[17]

**Table B.9:** Overview of the parameters affecting the continua. Explanation of the estimation may be found in the main text.

Continuum	Parameter	Notation	Value	Unit	Source

Nutrients	Diffusion	*D_n_*	0.833	*μm* ^*3*^ */min*	[17]
Nutrients	Decay	$\lambda_{n}$	0.00001	*min* ^−1^	[17]

VEGF	Diffusion	*D_v_*	0.175	*μm* ^*3*^ */min*	−
VEGF	Decay	$\lambda_{v}$	0.00001	*min* ^−1^	−

DOX	Diffusion	*D_d_*	0.708	*μm* ^*3*^ */min*	−
DOX	Decay	$\lambda_{d}$	0.0002	*min* ^−1^	−

TRA	Diffusion	*D_t_*	0.09	*μm* ^*3*^ */min*	−
TRA	Decay	$\lambda_{t}$	0.0	*min* ^−1^	−

## Appendix C. Large Scale Simulation - Mimicking the mirco-vasculature in the tumor environment

The initial structure of the micro-vasculature plays an important role. A denser vasculature may supply more nutrients and accelerate tumor evolution. However, data for the initialization is merely available. This section describes our approach to statistically mimic a realistic tumor micro-vasculature.

We first note that we do not consider a flow model; thus, connections between different segments of the vascular network do not affect the simulation outcome. Here, we place different vessel segments in the simulation space without explicitly considering their connections. Each segment consists of several agents that connect a start and end point and is further characterized by its diameter and length. Under these assumptions, we strive to find meaningful probability distributions to sample the diameter and length of the segments, as well as a reasonable number for the total number of segments.

In 1998, Secomb and coworkers [78] investigated the blood flow through the vasculature in the tumor region. The region’s volume is $V_{S}=(550\times 550\times 230)\mu m^{3}$, and they describe the different vessel segments by start and end point, diameter, and length. The data is available to the public and is the base for our statistical arguments.

As we ignore the connections, we neglect the start and end point information and focus on the data for diameter and length. We first compute the Pearson, Spearman, and Kendall-Tau correlation coefficients and obtain the values 0.35, 0.10, and 0.06, respectively. We conclude that treating the diameter and vessel length as independent random variables here is justified. To find a suitable probability density function (PDF), we fitted 95 PDFs to the data and chose the one yielding the most significant p-value for the Kolmogorov-Smirnov test. We find that the diameter is well described by a generalized extreme value continuous random variable, while a Wald continuous random variable better describes the length. The data and the fitted PDFs are displayed in Fig. C.11.

Before sampling from the PDFs, we truncate the Wald distribution, i.e. we require a minimum segment length of $l_{min}=100\mu m$. By computing the integral

(C.1)
$$\zeta =\int_{-\infty }^{l_{min}}\;p_{w}\left(x,\Theta_{w}\right)dx,$$

where $\Theta_{w}$ denotes the fitted parameters of the distribution, we obtain the ratio of samples that we ignore. Numerically, we evaluate the integral with an adaptive integration rule (Gauss-Kronrod 21-point) and obtain ζ≈0.78. In total, Secomb’s data contains $N_{S}=104$ vessel segments. According to our previous considerations, we initialize our simulation volume V with

(C.2)
$$N=N_{S}\cdot (1-\zeta )\cdot \frac{V}{V_{S}}$$

vessel segments.

After evaluating Eq. C.1 and C.2), we perform the following steps to initialize the vessel segments. First, we sample the diameter $d_{v}$ from the generalized extreme value distribution and the length $l_{v}$ from the fitted, truncated Wald distribution. Second, we sample a random point marking the segment’s start in the simulation space. Third, we sample a random point on a sphere of radius $l_{v}$ defining the segment’s endpoint. If the endpoint ends up outside the simulation volume, we resample. Once the start point, the endpoint, and the diameter are defined, we fill the line with cylindrical agents of appropriate diameter. If the length $l_{v}$ is larger than 200μm, we add smooth fluctuations from the center line, realizing that longer micro-vessels are unlikely to be straight.

To verify the approach, we compute the volume fraction occupied by the vasculature as

(C.3)
$$\rho_{S}=\frac{1}{V_{S}}\cdot \sum_{i=1}^{N_{S}}V_{i}\;\text{and}\;\rho =\frac{1}{V}\cdot \sum_{i=1}^{N}V_{i},$$

where $V_{i}$ denotes the volume of a vessel. For Secomb’s data, $V_{i}$ is computed from the length and diameter; for the simulation, $V_{i}$ is the sum of the agent volumes. We find the values $\rho_{S}=0.014308$ and ρ=0.004559 showing that the simulated vascular density is roughly 30% of the one given in the data. Since the vasculature density increases during the simulation due to angiogenesis, it is reasonable to begin with a lower vascular density.
